# Birth then and now

**DOI:** 10.1186/1741-7015-6-8

**Published:** 2008-03-18

**Authors:** Judith Lumley

**Affiliations:** 1 Mother & Child Health Research, La Trobe University, Melbourne, Australia

## Abstract

Halfway through the 20^th ^century, views on pain in labour encompassed almost everything from women's self-blame to blaming nurses, midwives, doctors or partners for 'bad experiences'. Soon after that, giving birth came to be seen – in some settings and by some caregivers – as a 'natural' and thus benign event which women could 'master'. In their recent systematic review of women's expectations and experiences of pain relief in labour, Joanne Lally, Madeleine Murtagh, Sheila Macphail, and Richard Thomson show that there is wide variation in women's expectations and knowledge about the first birth. This systematic review provides us with a strong basis for reflection and action.

## Commentary

My grandmothers, each of whom had seven surviving offspring, never discussed labour and never mentioned the pain of birth. This was not a heroic cover-up; their experiences of giving birth, at the beginning of the 20^th ^century, involved chloroform and they had hazy recollections and confused memories of what had happened during their labours and births. The other experience that contributed to women's reticence about their experiences of labour and birth was the high rate of fetal and infant mortality in those times. There were great wells of sadness about the infants who did not survive, even among families who had barely enough to 'keep the wolf from the door'.

Halfway through the 20^th ^century, views on pain in labour encompassed almost everything from women's self-blame to blaming nurses, midwives, doctors or partners for 'bad experiences'. Soon after that, giving birth came to be seen – in some settings and by some caregivers – as a 'natural' and thus benign event which women could 'master' or be trained to master. The shift of emphasis at that time included equally strong statements and beliefs about women's 'natural' capacity to give birth, including giving birth without pain. Formal preparation for birth began to be developed as the way of managing or coping with labour and birth in a variety of settings. There was a great diversity of methods and approaches, with some involving an active role for women's partners. Those providing direct care to women in labour, mostly the midwives and nurses, sometimes had a difficult time dealing with the concerns of labouring women, fathers, obstetricians, junior hospital staff, student midwives and student doctors.

Preparation and training for childbirth seemed to come into its own close to the time when the expansion of interventions in labour and birth was taking off. This made it increasingly likely that conflict between women themselves and those looking after women before or during labour would be exacerbated. The disagreements ranged from the best place for birth (home, 'homelike', labour ward, operating theatre) to the vexed question about who could be 'appropriate caregivers'. In some places there was a dispute as to who could be 'permitted' to be with the woman in labour, providing her with familiar faces and the promise of support. As hospital staff members – then and now – see caring for the mother as a key part of their role, disagreements between staff members and the woman's chosen carers about appropriate care were sometimes difficult to avoid. There was wide variation in women's expectations and knowledge about the first birth, just as there was marked variation between hospitals in policies and interventions. Underlying philosophies and practices contributed to these differences, though this was rarely explicit.

The recent systematic review of women's expectations and experiences of pain relief in labour carried out by Joanne Lally, Madeleine Murtagh, Sheila Macphail and Richard Thomson [1] provides us with a strong basis for reflection and action. Its focus is to review women's expectations and of pain and pain relief during labour and also the extent to which women were involved in the decision making process during labour. One of the strengths of their systematic review is the thoughtful and thorough selection of databases, going beyond the standard sources for medical, midwifery, nursing, and allied health, to sociological abstracts, PsychINFO and the midwifery-led collection of information and resources, MIDIRS. Another of the strengths is the inclusion of qualitative research papers. Thus, their analysis of the qualitative papers used the Critical Skills Programme (CASP), which was designed as an appraisal tool for qualitative research [2]. There were 346 quantitative and qualitative papers identified through searches but the reviewing process identified only 32 as meeting the criteria. Of these, 13 were qualitative and 19 quantitative. For comparison, there were 3630 titles and abstracts available for a recent systematic review of low-moderate prenatal alcohol exposure, with only 46 papers finally included [3].

Thomson and colleagues identified four key themes in these 32 papers: the level of pain, the type of pain, pain relief and women's involvement in decision-making and control. A consistent finding in the systematic review was that women underestimated the amount of pain they would experience. It was these mixed feelings that contributed to the title of the review: *More in hope than expectation*. That phrase might well be a good starting place for any mother, – first-time or not – since the experience of birth is likely to be rather different each time.

An alternative picture was also identified by the review, one which included the concept that pain in labour is different from pain associated with an illness. This interpretation offers the promise of some respite from severe pain during labour, but this is not necessarily feasible The variability in pain during labour may also offer some respite but if the variability of pain is high, marked variation in pain may be a mixed blessing.

I was very keen to see how the research team had assessed women's involvement in the decision-making process. An unexpected finding, to my mind, is that women who already had at least one child were more involved and interested in decision-making. However, having read in detail a number of the papers cited I can see how that came about. Women having their first child – rather than a second child – were described as 'concentrating on controlling their emotions, rather than being involved in decision making'. Birth plans turned out to 'give women an opportunity to consider and evaluate the options before labour began', a very useful contribution, but not one which will necessarily make a difference. One view – from a senior researcher was that "if women expect the worst pain imaginable they will end up having a painful, negative experience, in contrast to women whose view was more optimistic" This opinion is one that could be tested and it might be a useful piece of research.

Thus, the most important findings of the review are summarised in its last three paragraphs. As the authors state, 'Women may have ideal hopes of what they would like to happen, but they need to be educated or informed to ensure that they are prepared for what might actually happen and give them the tools to deal with this.' Implementation of the conclusions has the potential to make substantial improvements in understanding the many and diverse problems associated with the mismatch between labouring women's expectations and experiences, and may result in more responsive care.

## Pre-publication history

The pre-publication history for this paper can be accessed here:


